# Supplementary figures and images for: Comparative Molecular and Immunoregulatory Analysis of Extracellular Vesicles from Candida albicans and Candida auris
Source: mSystems. 2021 Aug 24;6(4):e00822-21. doi: 10.1128/mSystems.00822-21 (PMC8407381; doi:10.1128/mSystems.00822-21)

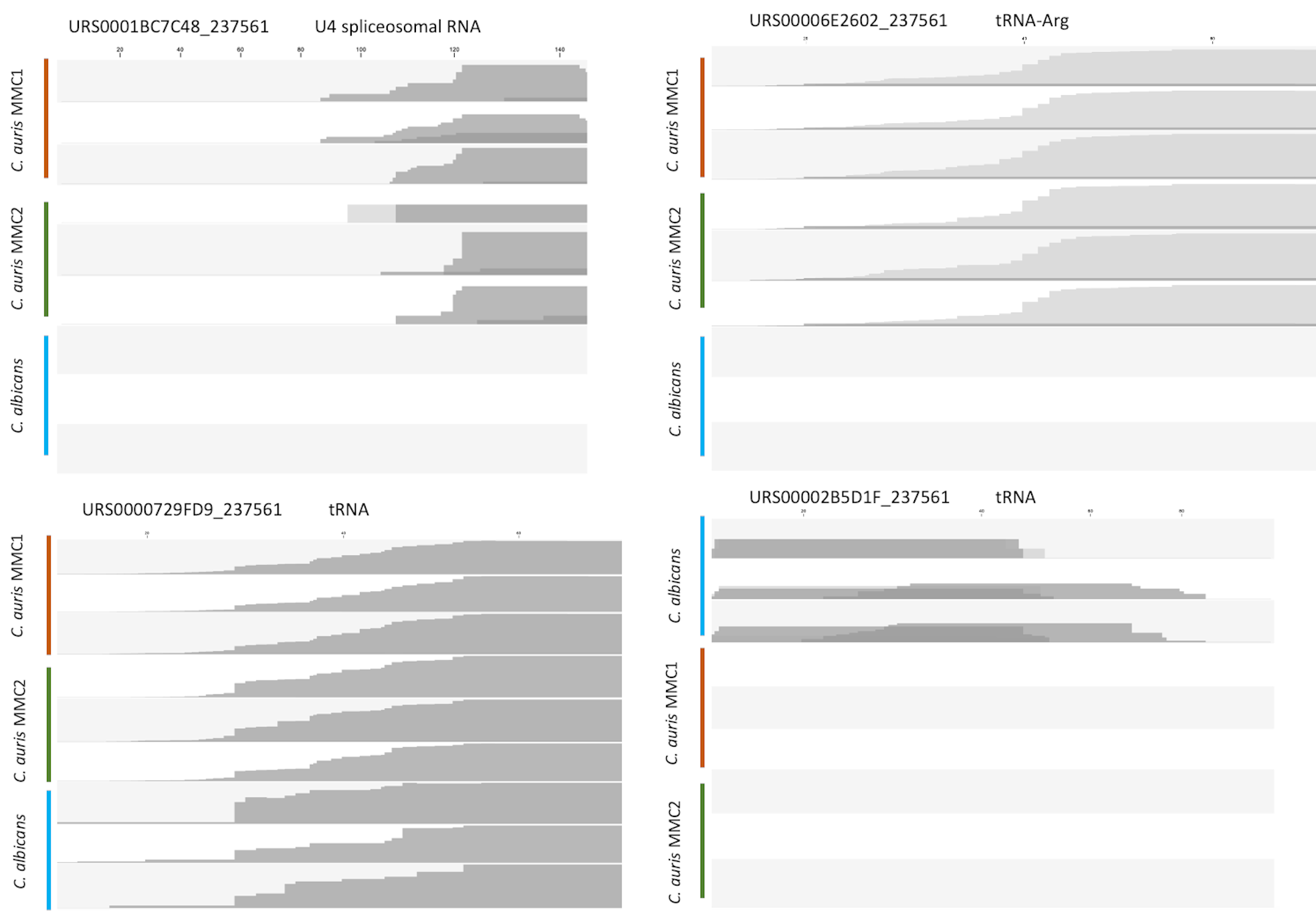

Supplement: FIG S1 [file msystems.00822-21-sf001.tif]

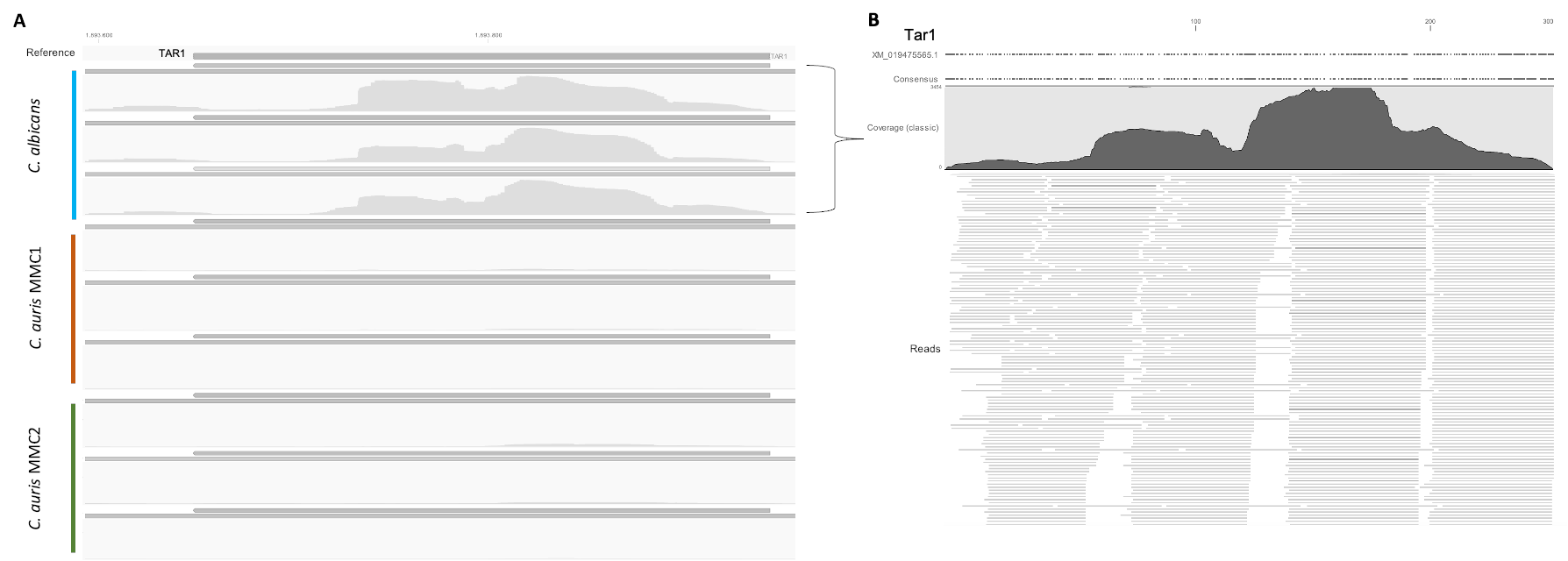

Supplement: FIG S2 [file msystems.00822-21-sf002.tif]
